# Cancer incidence and mortality among the Métis population of Alberta, Canada

**DOI:** 10.3402/ijch.v75.30059

**Published:** 2016-02-01

**Authors:** Diana C. Sanchez-Ramirez, Amy Colquhoun, Sara Parker, Jason Randall, Lawrence W. Svenson, Don Voaklander

**Affiliations:** 1 Injury Prevention Centre, School of Public Health, University of Alberta, Edmonton, AB, Canada; 2 Surveillance and Assessment Branch, Alberta Health, Edmonton, AB, Canada; 3 Métis Nation of Alberta, Edmonton, AB, Canada; 4 Department of Community Health, College of Medicine, Faculty of Health Sciences, University of Manitoba, Winnipeg, MB, Canada; 5 Department of Community Health Sciences, University of Calgary, Calgary, AB, Canada

**Keywords:** Aboriginals, Canada, cancer, incidence, Métis, mortality

## Abstract

**Background:**

Cancer has been identified as a major cause of morbidity and mortality in Canada over the last decade. However, there is a paucity of information about cancer patterns in Aboriginal people, particularly for Métis. This study aims to explore cancer incidence and mortality burden among Métis and to compare disease estimates with non-Métis population.

**Methods:**

This population-based descriptive epidemiological study used cancer incidence and mortality data from 2007 to 2012 obtained from Alberta Health Care Insurance Plan (AHCIP) – Central Stakeholder Registry – and Alberta Cancer Registry (ACR). To identify cancer cases in Métis, the ACR was linked with the Métis Nation of Alberta (MNA) Identification Registry. In Métis and non-Métis people, age-standardized cancer incidence and mortality rates were estimated and subsequently compared between both groups.

**Results:**

A higher incidence of bronchus/lung cancer was found among Métis men compared with their non-Métis counterparts (RR=1.69, CI 1.28–2.09; p=0.01). No other statistically significant differences in cancer incidence or mortality were found between Métis and non-Métis people living in Alberta over the course of the 6 years studied.

**Conclusions:**

Overall incidence and mortality associated with cancer were not higher among Métis people compared with non-Métis people. However, special efforts should be considered to decrease the higher incidence of bronchus/lung cancer in Métis men. Further development and maintenance of new and existing institutional collaborations are necessary to continue cancer research and health status surveillance in Métis population.

The term Métis is currently used to describe a group of Aboriginal people in Canada with mixed First Nations and European heritage, who have their own distinct culture and traditions. In 2003, the Supreme Court of Canada confirmed that Métis are a rights-bearing Aboriginal people. The components of a Métis definition for the purpose of claiming Aboriginal rights under section 35 of the Constitution Act 1982 are self-identification as a member of a Métis community, ancestral connection to the historic Métis community whose practices ground to right in question and acceptance by the modern community with continuity to the historic Métis community. The Métis National Council defines Métis as “a person who self-identifies as a Métis is distinct from other Aboriginal peoples, is of historic Métis Nation ancestry and is accepted by the Métis Nation” (1). A person who fulfils these requirements can apply to obtain Métis membership in the province within which they reside.

Data from the National Household Survey show that 451,795 of Canadians who reported Aboriginal ancestry identified themselves as Métis in 2011. They represented 32.3% of the total Aboriginal population and 1.4% of the total Canadian population.

Although numerous studies on health in Aboriginal populations exist, only a few differentiate findings between the 3 groups recognized in the Canadian Constitution as Aboriginal peoples: Métis, Inuit and First Nation populations (2,3). Moreover, Métis have typically been underrepresented in health research studies compared with other Canadian Aboriginal populations (3,4), partly due to a limited ability to identify Métis people within existing administrative health databases. Consequently, further studies, which characterize the health of Métis people, are required to prioritize health problems, to develop relevant policy options and to design and implement effective culturally specific strategies/interventions suitable to the needs of this population. Currently, the Métis Nation of Alberta (MNA) maintains a registry that permits the identification of Métis people living in Alberta. The linkage of this MNA membership registry to existing disease registries and administrative health databases offers an opportunity to better understand the health and health service use of this population.

Cancer is the leading cause of death in Canada, responsible for nearly 30% of all deaths (5). According to the Canadian Cancer Society, almost half of all Canadians will develop cancer in their lifetime, and a quarter of all of them are expected to die from this disease. Ageing and growing population are among the main factors explaining the increase in the number of new cases of cancer over the past few decades. As has occurred in the general Canadian population, chronic diseases including cancer have surpassed infectious diseases as major causes of morbidity and mortality among Aboriginal people over the last decades (6). Results from an 11-year follow-up study reported cancer as the first and second cause of death among Métis women (33% of total deaths) and men (26%), respectively (7). Previous studies have shown that overall incidence of all cancers combined is not higher among Aboriginal people compared with non-Aboriginal people (8,9). However, there is a paucity of information available about cancer patterns among Métis people in comparison with the general Canadian population. Therefore, the aim of this study is to provide information regarding cancer incidence and mortality burden among those registered with the MNA and to compare them with the general population in Alberta.

## Methods

### Data sources

This population-based descriptive epidemiological study used cancer incidence and mortality data from 2007 to 2012 obtained from the Alberta Ministry of Health. The target population included all people living in Alberta during the study period, without age restrictions. Through the Alberta Health Care Insurance Plan (AHCIP), Alberta Health assigns a unique personal health number (PHN) to all Albertans. This lifetime identifier, which is captured on all health care administrative records when people access hospital and/or medical services, allows for deterministic linkage between databases. An encrypted PHN was used for data linkage in the present study. Population estimates were obtained from the MNA Identification Registry and the AHCIP – Central Stakeholder Registry, for Métis and non-Métis people, respectively. Cancer incidence and mortality data were obtained from the Alberta Cancer Registry (ACR), maintained by Alberta Health Services. Ethical approval for this multi-year, retrospective review of Alberta data was obtained from the Health Research Ethics Board (HREB) at the University of Alberta.

### Cancer cases

All invasive incident cancer cases diagnosed between 2007 and 2012, excluding non-melanoma skin cancers, were included. To identify cancer cases in the Métis population, the MNA Identification Registry was linked with the ACR. The number of cases observed over the study period was compiled by Métis status, sex and cancer site as classified by the International Classification of Diseases for Oncology (ICD-O-3): bronchus/lung (C34), breast (C50), colorectal (C18–C20) and prostate (C61). For cancer sites with a small number of cases in the Métis population, the number of cases observed was reported by larger topographical category: head and neck (C00–C14, C30–C32), digestive organs (excluding colorectal; C15–C17, C21–C26), female genital organs (C51–C58), urinary tract (C64–C68), hematopoietic and reticuloendothelial systems (C42, C77) and all other topographies. All deaths due to cancer within the study period were included; cancer sites for deaths were classified using ICD-10-CA (10).

### Statistical analysis

For the 6-year study period (2007–2012), standardized cancer incidence (SIRs) and mortality (SMRs) rates were estimated by Métis status, sex, the top 4 cancer sites (lung, colorectal, breast and prostate) and all cancers combined. Rates were age standardized using 5-year age groups, standardized to the 1991 Canadian population. To assess rates over time, a 3-year moving average was used to minimize fluctuations resulting from small numbers of cases in the Métis population. Rate ratios (RR) and their CIs were estimated to compare rates between Métis and non-Métis populations. Statistical significance was accepted at p-values <0.05. Data analysis was performed using SAS^®^ 9.3 software (SAS Institute, Cary NC).

## Results

### Descriptives

An annual average of 23,807 Métis people (51% women) and 3,675,388 non-Métis people (49% women) living in Alberta between 2007 and 2012 were included in the present study. Métis people were slightly younger, with a smaller percentage aged 55 years and older, compared with the non-Métis population (16% vs. 21%). A larger percentage (37%) of Métis people were living in a rural area, defined as towns and municipalities outside the commuting zone of larger urban centres, compared with the non-Métis population (23%) (Table I).

**Table I:** Study population

Alberta, Canada, 2007–2012				

	Average annual population over the 6-year study period		New cases of cancer between 2007 and 2012 (excluding non-melanoma skin cancer)	
		
	Métis	Non-Métis	Métis	Non-Métis
Characteristics				
N	23,793	3,676,253	440	90,762
Women, n (%)	12, 070 (50.7)	1,808,291 (49.2)	227 (51.6)	43,747 (48.2)
Men, n (%)	11,723 (49.3)	1867962 (50.8)	213 (48.4)	47,015 (51.8)
aRural area, n (%)	8,846 (37.2)	828,640 (22.5)	173 (39.3)	22,827 (25.2)
Age groups (years), n (%)				
<24	9,024 (37.9)	1,203,767 (32.7)	10 (2.3)	1,449 (1.6)
25–34	3,532 (14.8)	591,694 (16.1)	17 (3.9)	2,200 (2.4)
35–44	3,613 (15.2)	537,443 (14.6)	21 (4.8)	4,723 (5.2)
45–54	3,870 (16.3)	563,732 (15.3)	80 (18.2)	13,225 (14.6)
55–64	2,248 (9.4)	390,341 (10.6)	135 (30.7)	21,756 (24.0)
65–74	1,128 (4.7)	210,866 (5.7)	120 (27.3)	22,722 (25.0)
75 +	378 (1.6)	178,430 (4.9)	57 (13.0)	24,687 (27.2)
Types of cancer, n (%)				
Bronchus/lung	–	–	60 (13.6)	11,348 (12.5)
Colorectal	–	–	53 (12.0)	10,916 (12.0)
Female breast cancer	–	–	76 (17.3)	12,916 (14.2)
Prostate	–	–	53 (12.0)	13,266 (14.6)
Head and neck	–	–	21 (4.8)	2,611 (2.9)
Digestive organs (excluding colorectal)	–	–	32 (7.3)	7,494 (8.3)
Female genital organs	–	–	29 (6.6)	5,184 (5.7)
Urinary tract	–	–	36 (8.2)	5,071 (5.6)
Hematopoietic and reticuloendothelial systems	–	–	41 (9.3)	10,520 (11.6)
Other	–	–	39 (8.9)	11,436 (12.6)
Mortality associated with cancer between 2007 and 2012, *n*	118	34,935	–	–

a Rural status information was missing for some non-Métis individuals (on average, 154 individuals over the study period).

### Incidence of cancer

Over the 6 years of the study, there were 440 new cases of cancer diagnosed in Métis and 90,762 new cancer cases diagnosed in the non-Métis population (Table I). The majority of new cases of cancers in both groups were diagnosed among people aged 55 years or older (71% of total cases in Métis; 76% in non-Métis). In Métis and non-Métis people, when both men and women were analysed together, the most common type of cancer diagnosed was bronchus/lung cancer followed by colorectal cancer (Table I). For women, breast cancer was the most common type of cancer, representing 34% of all cancers diagnosed in Métis women (30% in non-Métis). Prostate cancer was the most common cancer diagnosed in men, 25% and 28% of all cancers in Métis and non-Métis men, respectively. Cancers of the digestive organs (excluding colorectal), as well as those of the hematopoietic and reticuloendothelial systems, were among the less common cancers observed in the Métis. Topographies of incident cancer cases by broad anatomical group categories are shown in Table II.

**Table II:** Topographies of incident cancer cases and deaths observed in the Métis population by broad anatomical group, Alberta, 2007–2012

	Topography	
	
Anatomical category	Incident cancer cases	Cancer deaths
Head and neck	Lip; base of tongue; tongue, other and unspecified; palate; mouth, other and unspecified; tonsil; oropharynx; nasopharynx; lip, oral cavity and pharynx, other and unspecified; accessory sinuses; larynx	–
Digestive organs	Oesophagus; stomach; small intestine; anus and anal canal; liver and intrahepatic bile ducts; gallbladder; biliary tract, other and unspecified; pancreas	Stomach; small intestine; colorectal; liver and intrahepatic bile ducts; gallbladder; pancreas
Female genital organs	Vulva; vagina; cervix uteri; endometrium; other uterus and uterus, NOS; ovary	–
Urinary tract	Kidney; renal pelvis; bladder	–
Hematopoietic and reticuloendothelial systems	Non-Hodgkin lymphoma; leukaemia; multiple myeloma and plasmacytoma; other hematopoietic and reticuloendothelial	Non-Hodgkin lymphoma; leukaemia; multiple myeloma and plasmacytoma; other hematopoietic and reticuloendothelial
Other	Bones, joints and articular cartilage of limbs; connective, subcutaneous and other soft tissues; melanoma of skin; penis; testis; brain; thyroid gland; other and ill-defined sites; unknown primary	Base of tongue; tongue, other and unspecified; palate; tonsil; hypopharynx; lip, oral cavity and pharynx, other and unspecified; accessory sinuses; connective, subcutaneous and other soft tissues; retroperitoneum and peritoneum; cervix uteri; ovary; prostate gland; kidney; bladder; brain; thyroid gland; unknown primary

**Fig. 1:**
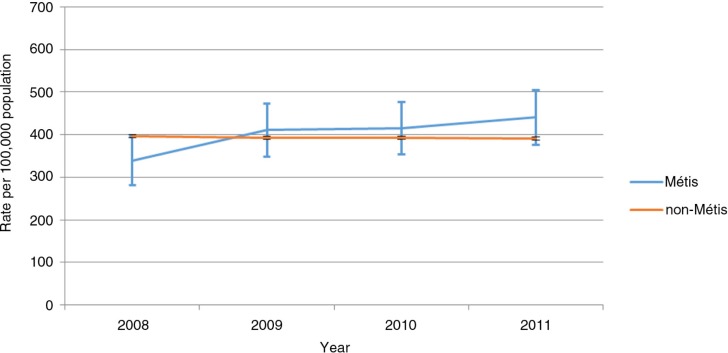
Age-standardized incidence rates (ASIR) for cancers in Métis and non-Métis people by year, Alberta. *3-year moving average. Rates in Métis people are based on a small number of cases and should be interpreted with caution.

Age-standardized incidence rates (ASIRs) for all cancers combined were fairly stable among the non-Métis over the study period, while ASIRs appeared to increase slightly over time in the Métis population (Fig. 1). Over the course of the 6 years, the ASIR for lung cancer was significantly higher in Métis compared with non-Métis people (RR=1.47, CI 1.14–1.80; p=0.02), particularly in men (RR=1.69, CI 1.28–2.09; p=0.01) (Table III). Though not statistically significant, the ASIR for prostate cancer was lower in Métis men compared with their non-Métis counterparts (RR=0.75, CI 0.46–1.04; p=0.05). ASIRs for colorectal cancer, for female breast cancer and for all cancers combined were not significantly different between Métis and non-Métis populations during the period studied.

**Table III:** Age-standardized incidence rates (ASIRs) for cancers in Métis and non-Métis people by sex, Alberta, calculated over a 6-year period (2007–2012)

		Métis		non-Métis			
					
Type of cancer	Sex	ASIR	95% CI	ASIR	95% CI	Rate ratio	p-values^b^
Bronchus/lung	Both sexes	74.5	50.0, 99.0	50.8	49.9, 51.8	**1.47**	**0.02**
	Males	95.5	57.1, 133.8	56.4	54.9, 57.9	**1.69**	**0.01**
	Females	50.0	22.3, 77.8	46.9	45.6, 48.1	1.07	0.82
Colorectal	Both sexes	48.7	33.8, 63.7	47.4	46.5, 48.3	1.03	0.86
	Males	61.8	38.7, 84.8	57.9	56.5, 59.4	1.07	0.74
	Females	34.8	16.4, 53.2	38.0	36.9, 39.1	0.92	0.75
Breast	Females	125.3	99.2, 158.3	105.5	103.7, 107.4	1.19	0.20
Prostate	Males	91.9	65.4, 118.3	122.7	120.5, 124.8	0.75	0.05
All cancers combined^a^	Both sexes	401.5	356.2, 446.8	393.0	390.4, 395.6	1.02	0.71
	Males	401.8	338.7, 464.9	436.4	432.4, 440.4	0.92	0.31
	Females	393.0	331.8, 454.3	360.7	357.3, 364.1	1.09	0.28

CI, confidence interval.

a Excluding non-melanoma skin cancer. Rates per 100,000 population

b differences in ASIRs were tested assuming independence of ASIRs and binomial variances in each age-specific stratum. Significant differences are given in boldface.

### Mortality related to cancer

Between 2007 and 2012, there were 118 deaths due to cancer in the Métis population; 34,935 non-Métis people died due to cancer over the same time period (Table I). The majority of cancer deaths were due to bronchus/lung cancer, representing 33% and 25% of total deaths in Métis and non-Métis people, respectively. Causes of cancer death also included breast cancer, as well as cancers of the digestive organs and hematopoietic and reticuloendothelial systems. Topographies of specific cancer sites within broader categories causing death in the Métis during the study period are shown in Table II.

Age-standardized mortality rates (ASMRs) associated with cancer were not statistically different between Métis and non-Métis people. However, the ASMR for breast cancer in Métis women was half of the rate estimated for their non-Métis counterparts (RR=0.50, CI 0–1.2; p=0.06) (Table IV).

**Table IV:** Age-standardized mortality rates (ASMRs) for cancers in Métis and non-Métis people, Alberta, calculated over a 6-year period (2007–2012)

	Métis		non-Métis			
				
Type of cancer	ASMR	95% CI	ASMR	95% CI	Rate ratio	p-values^a^
Bronchus/lung	51.6	30.6, 72.5	39.1	38.2, 39.9	1.32	0.18
Colorectal	9.8	2.6, 17.1	17.1	16.6, 17.7	0.57	0.14
Breast (females only)	9.7	2.8, 16.6	19.2	18.5, 20.0	0.50	0.06
Prostate (males only)	13.9	0.4, 27.5	21.9	20.9, 22.8	0.64	0.36
All cancers combined	131.8	101.4, 162.3	151.5	149.9, 153.1	0.87	0.24

CI, confidence interval. Rates per 100,000 population.

a Differences in ASMRs were tested assuming independence of ASMRs and binomial variances in each age-specific stratum.

## Discussion

This is the first study to describe cancer incidence and mortality in the Métis population of Alberta, Canada, and to compare estimates with the non-Métis populations within the same province. During the study period, bronchus/lung cancer incidence was substantially higher in Métis men compared with their non-Métis counterparts. However, incidence rates for other types of cancers in Métis people were not remarkably different from non-Métis people. Bronchus/lung cancer was the leading cause of cancer death in both populations. However, there were no significant differences in mortality associated with cancer between Métis and non-Métis people.

Our results correspond with previous studies carried out in Ontario and Quebec, which reported an overall similar risk of cancer between Aboriginal and non-Aboriginal populations (8,11). Former evidence has suggested a lower lung cancer incidence among Aboriginal people compared with the general population (12). However, evidence has shown that lung cancer rates among Aboriginal people have increased over time (13) and, as reported in the present study, exceeded the rate of the non-Aboriginal population. This phenomenon may be explained by greater exposure to risk factors such as smoking. Although data related to the prevalence of smoking in Métis people are not available, a previous report from the First Nations Information Governance Centre showed that prevalence of smoking in this Aboriginal group was more than double (57%) the prevalence of the general Canadian population around the same period of time (18%) (14,15).

Some limitations of the present study need to be considered. The Métis population was identified using the MNA identification registry. As this list is comprised only of Métis people who have registered with the MNA and met their membership requirements, it does not capture all people with Métis ancestry living in Alberta. Therefore, additional mechanisms should be explored to better identify the Métis population. Moreover, because the registered MNA population is small, there were a relatively small numbers of new cases of cancer diagnosed and cancer-related deaths in Métis compared with non-Métis people. Consequently, the rates in the Métis population are based on a small number of cases resulting in wide confidence intervals and should be interpreted with caution.

The main strength of the present study is the use of high-quality provincial health administrative and disease registry data. The ACR consistently receives a gold certification from the North American Association of Central Cancer Registries (NAACCR) for its completeness, timeliness, validity and comparability. Furthermore, another strength of this study is the collaboration among the Métis Nation of Alberta, University of Alberta and the provincial government. The valuable contributions of these institutions permitted the identification of MNA members in provincial administrative databases and disease registries, something not previously possible. However, further development and maintenance of the institutional collaborations are necessary to continue cancer research and health status surveillance over time among Métis population.

The estimates presented in the current study address the gaps in information available on cancer in Métis people. We hope that findings from this study can be used by health planners for the design and implementation of strategies directed to reduce cancer incidence and mortality in Métis people. In order to have a further understanding of cancer among Métis, the information provided in the present study should be complemented with further studies in cancer survival in Métis people and factors associated with it, such as specific risk factors and determinants of cancer in this population. In addition, cancer data should be available well into the future for further research and surveillance.

## Conclusions

Overall incidence and mortality estimates for most types of cancer were comparable among Métis and non-Métis populations. However, special efforts should be considered to decrease the higher incidence of bronchus/lung cancer in Métis men. Further development and maintenance of new and existing institutional collaborations are necessary to continue cancer research and health status surveillance in Métis population.
